# USP5 Suppresses Ferroptosis in Bladder Cancer Through Stabilization of GPX4

**DOI:** 10.3390/cimb47030211

**Published:** 2025-03-20

**Authors:** Caiying Liu, Yanong Deng, Liang Huang, Xinrui Nie, Yuxuan Jiang, Xia Zhang, Huihui Zhang

**Affiliations:** School of Medical Technology and Translational Medicine, Hunan Normal University, 371 Tongzipo Road, Yuelu District, Changsha 410013, China; 202220193564@hunnu.edu.cn (C.L.); 202470193818@hunnu.edu.cn (Y.D.); 202120193364@hunnu.edu.cn (L.H.); 202030193005@hunnu.edu.cn (X.N.); jiangyuxuan1246@hunnu.edu.cn (Y.J.)

**Keywords:** bladder cancer, USP5, GPX4, ferroptosis

## Abstract

USP5 has been proven to play an important role in the proliferation of bladder cancer (BC). In this study, we focused on investigating the molecular mechanism of ferroptosis induced by USP5 in bladder cancer. The role of USP5 in bladder cancer was evaluated using T24 wild-type cells (WT) and USP5 knockout (USP5^−/−^) by CCK8 and colony formation assays. The contents of ferrobivalent ions (Fe^2+^), reactive oxygen species (ROS), and malondialdehyde (MDA) were detected using a determination kit to observe the relationship between USP5 and ferroptosis. Furthermore, the molecular mechanism study was evaluated by employing Western blotting, co-immunoprecipitation, RT-qPCR, ubiquitination assays, etc. This study showed genetic ablation of USP5 significantly inhibited the viability and proliferation of bladder cancer cells. Genetic ablation of USP5 promoted increases in Fe^2+^ content, ROS, and MDA levels. The addition of erastin significantly increased the viability and proliferation of T24 USP5^−/−^ cells and significantly increased their ROS and MDA contents. We verified that USP5 deficiency led to a significant reduction in GPX4 protein levels and that the overexpression of USP5 could stabilize the GPX4 protein. Further studies showed that USP5 interacts with GPX4 and stabilizes GPX4 by inhibiting its ubiquitination These findings revealed USP5 inhibits ferroptosis in bladder cancer cells by stabilizing GPX4. The relationship between USP5 and ferroptosis could be a potential therapeutic target for bladder cancer.

## 1. Introduction

Bladder cancer (BC) is one of the most prevalent malignant tumors in the urinary system [1]. BC is an enormous social burden, with more than 610,000 new cases and more than 220,000 cancer deaths worldwide in 2022 [2]. The incidence rates exhibit considerable geographical variation and are influenced by factors such as environmental exposure, lifestyle choices, and genetic predispositions. For example, bladder cancer is notably more prevalent in males than in females, with a reported incidence ratio of approximately 34:10 [2]. The pathophysiology of BC involves complex interactions between genetic mutations, environmental factors, and cellular signaling pathways. The accumulation of genetic mutations leads to the dysregulation of critical cellular pathways, including those involved in cell cycle control, apoptosis, and DNA repair mechanisms [3]. Despite numerous studies, the full mechanism of BC development remains unknown.

Deubiquitinating enzymes (DUBs) are a class of proteases that play crucial roles in the regulation of protein stability and cellular homeostasis by removing ubiquitin from target proteins [4]. This deubiquitinating activity is essential for cellular processes, thus influencing various physiological and pathological conditions, including cancer, neurodegenerative diseases, and immune responses [5,6,7]. Emerging research indicates that DUBs are potential drug targets for cancer treatment [7,8]. Ubiquitin-specific protease 5 (USP5), a DUB family member, removes ubiquitin from the proximal end of the unanchored multiubiquitin chain [9]. Several studies have demonstrated that USP5 plays an important role in cancers, including liver, lung, breast, ovarian, and colorectal cancers [10,11,12,13,14]. Moreover, studies have shown that USP5 is significantly associated with several crucial molecules, such as p53 [15], FoxM1 [16], β-catenin [17], HDAC2 [11], and TUFM [10]. These findings suggest that USP5 could represent a potentially valuable therapeutic target in the treatment of cancer. Our previous study demonstrated that USP5 is overexpressed in bladder cancer and promotes cell proliferation, migration, and tumor formation [18]. However, the pathogenic mechanisms by which USP5 is involved in bladder tumourigenesis and progression need to be explored.

Ferroptosis is a form of regulated cell death characterized by the accumulation of lipid peroxides and iron-dependent reactive oxygen species (ROS) [19]. Comprehensive research has demonstrated that ferroptosis plays a critical role in tumors, suggesting new therapeutic opportunities for cancer treatment [20]. The Nrf2/HO-1/GPX4 signaling axis is one of the important pathways regulating ferroptosis; this axis can promote glutathione (GSH) synthesis, inhibit lipid peroxidation, and thus inhibit the occurrence of ferroptosis [21]. The selenoprotein GPX4 (glutathione peroxidase 4), originally called PHGPX (phospholipid hydroperoxidase glutathione peroxidase), is a major redox agent that uses glutathione as a reducing agent to remove lipid peroxidation products. In recent years, it has been recognized as a key regulator of ferroptosis that affects cell aging, tumourigenesis, and cell death. There is increasing evidence that targeting GPX4-induced ferroptosis is a promising treatment strategy for diseases, especially cancer [22]. The stability and function of GPX4 are tightly regulated by post-translational modifications, particularly ubiquitination and deubiquitination processes [23,24]. However, the regulation of GPX4 by USP5 needs to be explored.

In this study, we found that USP5 deficiency inhibits the proliferation of T24 bladder cancer cells. Mechanistically, we discovered that USP5 deficiency promotes ferroptosis. Further investigation revealed that USP5 binds to and stabilizes GPX4 by mediating its deubiquitination. Collectively, our findings reveal a novel mechanism by which USP5 regulates ferroptosis through GPX4 stabilization, highlighting its critical role in bladder cancer development and progression. These results provide new insights into the molecular mechanisms underlying ferroptosis regulation and suggest that USP5 is a potential therapeutic target for bladder cancer treatment.

## 2. Materials and Methods

### 2.1. Antibodies and Plasmids

For protein detection, commercially available antibodies were used: rabbit polyclonal anti-USP5 (10473-1-AP, Proteintech, Wuhan, China); mouse polyclonal GPX4 antibody (67763-1-Ig, Proteintech, Wuhan, China); mouse polyclonal anti-HA (M180-3, MBL, Tokyo, Japan, 1:5000 dilution); mouse polyclonal anti-Flag (M185-11R, MBL, Japan, 1:5000 dilution); mouse polyclonal anti-Myc (M192-3, MBL, Japan, 1:5000 dilution); mouse polyclonal anti-GAPDH (ANT011, AntGene, Wuhan, China, 1:5000 dilution); HRP-labelled goat anti-mouse IgG (H + L) (A0216, Beyotime, Shanghai, China, 1:5000 dilution); and HRP goat anti-rabbit IgG (H + L) (ANT020, AntGene, Wuhan, China, 1:5000 dilution).

The plasmids pHAGE-3 × FLAG-GPX4 (FLAG-GPX4), pHAGE-3 × HA-USP5 (HA-USP5), and pHAGE-3 × HA-USP5 C335A (HA-USP5 C335A) were constructed according to the methods in the “Molecular Cloning Experiment Guide”.

### 2.2. Cell Culture and Cell Lines

The cell lines utilized in this study were purchased from ATCC. For HEK293T cell culture, Dulbecco’s modified Eagle’s medium (DMEM; HyClone, Logan, UT, USA) was used as the growth substrate. McCoy’s 5a medium (Basal Media, Shanghai, China) served as the culture matrix for T24 cells. According to ATCC, T24 cells’ doubling time is 19 h. The media were supplemented with 10% fetal bovine serum (FBS; Gibco, Hercules, CA, USA) and 1% penicillin/streptomycin (Gibco, Hercules, CA, USA). All the cells were cultured at 37 °C in a 5% CO_2_ incubator. No mycoplasma contamination was detected.

HEK293 cells were used for protein interaction, protein stability, and ubiquitination. T24 cells were used to generate knockout cell lines and evaluate phenotypic changes.

### 2.3. Cell Proliferation and Colony Formation

Cell proliferation was assessed using the CCK-8 assay (BS350B, Biosharp, Hefei, China) as we previously described [18]. Briefly, cells were plated at a density of 1 × 10^3^ cells/well in 96-well plates. Following the incubation period, 10 µL of CCK-8 reagent was mixed with 100 µL culture medium and added to each well, followed by a 1-hour incubation at 37 °C. Absorbance was quantified at 450 nm using a spectrophotometer. For colony formation assays, 6 × 10^2^ cells were seeded in 6-well plates and cultured for 14 days. The colonies were fixed and stained with 0.025% crystal violet, followed by digital imaging using a scanner.

### 2.4. Determination of Intracellular ROS

Intracellular ROS levels were measured via a ROS determination kit (R6033, UElandy, Suzhou, China) following the manufacturer’s instructions. The cells were seeded into 6-well plates (1 × 10^5^ cells/well). After 24 h, the cells were treated with erastin for 48 h. The cells were subsequently labeled with 2′,7′-dichlorofluorescein diacetate (DCFH-DA) at 37 °C for 30 min. Finally, the intracellular ROS level was detected by flow cytometry.

### 2.5. Measurement of the Intracellular Fe^2+^ and MDA Contents

The level of intracellular Fe^2+^ ions was measured with a Cell Ferrous Iron (Fe^2+^) Fluorometric Assay Kit (MA0647, MeilunBio, Dalian, China) according to the manufacturer’s instructions. The cells were seeded at a density of 1  ×  10^5^ cells/mL in a 12-well plate. The cells were treated with erastin for 48 h. The medium was removed, and the cells were washed with PBS 2–3 times. Next, 100 μL of staining working solution was added, and the mixture was incubated at 37 °C in a 5% CO_2_ incubator for 20–60 min. Finally, the fluorescence was immediately detected by a fluorescence microscope (DMi8, Leica, Wetzlar, Germany) with an excitation wavelength of 543 nm and an emission wavelength of 580 nm.

The cells in each group were collected, and the MDA levels were measured via a malondialdehyde (MDA) assay kit (TBA method) (A003-1-2, Nanjing Jiancheng Bio, Nanjing, China). The steps were carried out according to the manufacturer’s instructions.

### 2.6. Western Blot Analysis

The cells were lysed with SDS lysis buffer (62.5 mM Tris-HCl (pH 6.8), 2% SDS, and 10% glycerol) at 95 °C for 10 min. Total protein was separated by 10% SDS‒PAGE and transferred to PVDF membranes (IPVH00010; Millipore, Billerica, MA, USA). The membranes were blocked with 5% skim milk for 1 h. The membranes were incubated with primary antibodies overnight at 4 °C. The next day, the membranes were washed in TBST and then incubated with HRP-labeled secondary antibodies at room temperature for 1 h. A Tanon 5500 chemiluminescence image analysis system (Tanon, Shanghai, China) was used to evaluate the chemiluminescence of the protein bands. GAPDH was used as the internal control.

### 2.7. Coimmunoprecipitation

Cell lysate was prepared using NP-40 lysis buffer (N8032, Solarbio, Beijing, China) supplemented with protease inhibitor cocktails. The lysates were centrifuged at 12,000 rpm for 10 min at 4 °C. The supernatant of the lysate was incubated with the appropriate antibody and protein A/G agarose beads (SM005002, SMART Life Sciences, Changzhou, China) overnight at 4 °C. The following day, the agarose beads were rinsed with lysis buffer 3 times. The immunoprecipitated proteins were heat-denatured (95 °C, 10 min) in 2 × SDS‒PAGE loading buffer and separated using 12% SDS-polyacrylamide gels.

To detect ubiquitination, MG132, a proteasome inhibitor, was added to the cells to prevent the degradation of ubiquitinated proteins.

### 2.8. Real-Time Quantitative PCR

According to the manufacturer’s protocol, an RNAeasy^TMd^ extraction kit (which was used to extract RNA), Evo M-MLV reverse transcription reagent premix (AG11705, Accurate Biology, Changsha, China), and ChamQ Universal SYBR qPCR Master Mix (Q77, Vazyme Biotech, Nanjing, China) were used for PCR detection. The primers used are shown in Table 1.

### 2.9. Statistical Analysis

Two-tailed unpaired Student’s *t* test was used to compare two groups of data. One-way ANOVA was used to compare multiple groups of data. A *p* value of less than 0.05 was considered significant.

## 3. Results

### 3.1. USP5 Deficiency Inhibits Cell Viability and Proliferation

**Figure 1:**
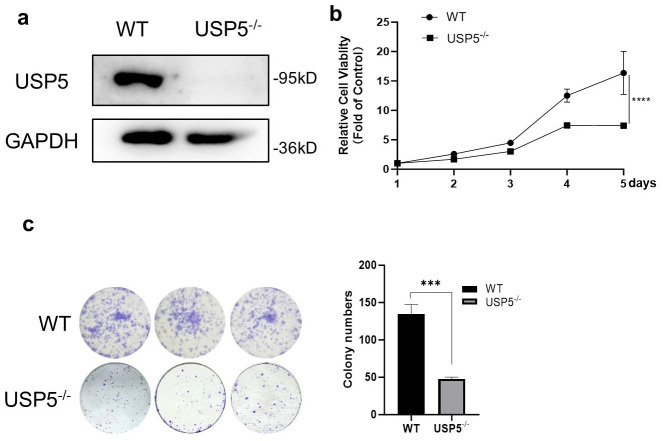
USP5 deficiency inhibits cell viability and proliferation. (**a**) Western blot detection of USP5 protein levels in cells (WT vs. USP5^−/−^), normalized to endogenous GAPDH expression. (**b**) Cell proliferation analysis using CCK-8 reagent, with absorbance measurements recorded daily for 5 consecutive days (*n* = 6 biological replicates). (**c**) Assessment of cellular viability through colony formation assay: left panel displays crystal violet-stained colonies (×4), while right panel presents quantitative colony counts (*n* = 3). The data (mean ± SEM) are representative of 3 independent experiments. Statistical significance was analyzed by ANOVA or Student’s *t* test. *** *p* < 0.001, **** *p* < 0.0001.

Clustered regularly interspaced short palindromic repeats (CRISPR)/Cas9 technique was used to construct bladder cancer T24 USP5-deficient cells (USP5^−/−^) [18]. Immunoblot analysis confirmed the successful ablation of USP5 protein expression (Figure 1a). Functional assessment of USP5 in cell proliferation revealed significantly reduced growth rates in knockout cells compared to wild-type controls, as determined by CCK-8 assays (Figure 1b). Consistent with these findings, the colony formation ability was markedly reduced in USP5^−/−^ cells, as evidenced by colony formation assays (Figure 1c). Together, these experimental observations indicate that USP5 plays a crucial role in maintaining the proliferative and survival capacity of T24 bladder cancer cells.

### 3.2. USP5 Deficiency Promotes Ferroptosis in T24 Cells

**Figure 2:**
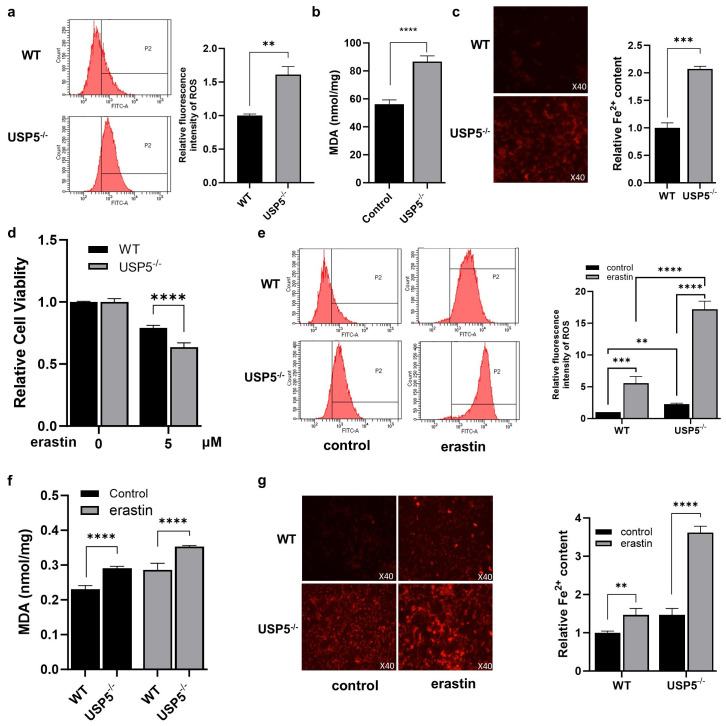
USP5 deficiency promotes ferroptosis in T24 cells. (**a**) ROS content of WT and USP-deficient T24 cells determined via the DCFH-DA fluorescent probe method. (**b**) MDA content of WT and USP-deficient T24 cells determined via kits (*n* = 3). (**c**) The Fe^2+^ content of WT (top) and USP5-deficient T24 cells (bottom) determined via kits (40× magnification). (**d**) The relative cell viability of WT and USP-deficient T24 cells was detected after erastin addition (*n* = 3). (**e**–**g**) The levels of ROS, MDA, and Fe^2+^ resulting from USP5 deficiency were detected after erastin addition. The data (mean ± SEM) are representative of 3 independent experiments. Statistical significance was analyzed by ANOVA or Student’s *t* test. ** *p* < 0.01, *** *p* < 0.001, **** *p* < 0.0001.

Ferroptosis has emerged as a critical regulator of cancer. Previous studies revealed that USP5 suppresses ferroptosis and promotes the malignant progression of tumors. We next explored whether USP5 deficiency affects ferroptosis in T24 cells. As expected, the ROS level was greater in USP5^−/−^ cells than in WT cells (Figure 2a). As shown in Figure 2b, USP5 deficiency increased MDA production. Consistently, the Fe^2+^ level was greater in USP5-deficient cells than in WT cells (Figure 2c). In addition, we investigated whether USP5 deficiency had an impact on cell viability upon ferroptotic stimulation. As expected, erastin treatment significantly reduced the viability of T24 cells, which was aggravated by USP5 deficiency (Figure 2d). Accordingly, erastin-induced increases in ROS, MDA, and Fe^2+^ levels were increased by USP5 deficiency (Figure 2e–g). These results indicate that USP5 deficiency promotes ferroptosis.

### 3.3. USP5 Modulates Ferroptosis by Targeting GPX4

**Figure 3:**
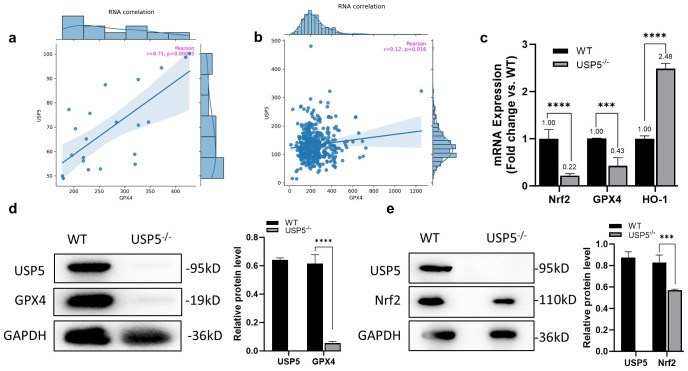
USP5 modulates ferroptosis by targeting GPX4. (**a**) Association between USP5 and GPX4 RNA levels in bladder tissue on the basis of GTEx data (left) and between USP5 and GPX4 RNA levels in bladder cancer tissue on the basis of TGCA data (right). (**b**) USP5 and GPX4 RNA levels in bladder cancer tissues were determined on the basis of TGCA data. (**c**) RT‒qPCR was used to detect the GPX4, NRF2, and HO-1 mRNA levels in USP5-deficient cells (*n* = 3). GAPDH was used as an internal control for normalization. (**d**) Immunoblot analysis of GPX4 expression profiles in WT versus USP5^−/−^ T24 cells, using GAPDH for normalization. (**e**) Comparative assessment of Nrf2 protein abundance between WT and USP5^−/−^ T24 cells, with GAPDH serving as internal reference. The data are representative of 3 independent experiments. Statistical significance was analyzed by ANOVA or Student’s *t* test., *** *p* < 0.001, **** *p* < 0.0001.

We next aimed to elucidate the possible mechanisms by which USP5 affects ferroptosis. Considering that GPX4 is a key factor in the ferroptosis signaling pathway, we analyzed the correlation between USP5 and GPX4 in bladder cancer via the FerrDb database. The results revealed a significant positive correlation between USP5 and GPX4 RNA levels in bladder tissue on the basis of the GTEx data and a positive association between USP5 and GPX4 RNA levels in bladder cancer tissue on the basis of the TGCA data (Figure 3a,b). As shown in Figure 3c, USP5 deficiency significantly reduced GPX4 mRNA levels. In addition, the mRNA levels of NRF2 and HO-1 were affected by USP5 deficiency. We examined whether the absence of USP5 affected the protein levels of GPX4 and NRF2. The results revealed that the protein levels of GPX4 and NRF2 were decreased in USP5^−/−^ cells (Figure 3c,d). Interestingly, the absence of USP5 led to a significant reduction in the GPX4 protein. These results show that USP5 modulates ferroptosis by targeting GPX4.

### 3.4. USP5 Interacts with and Stabilizes GPX4

**Figure 4:**
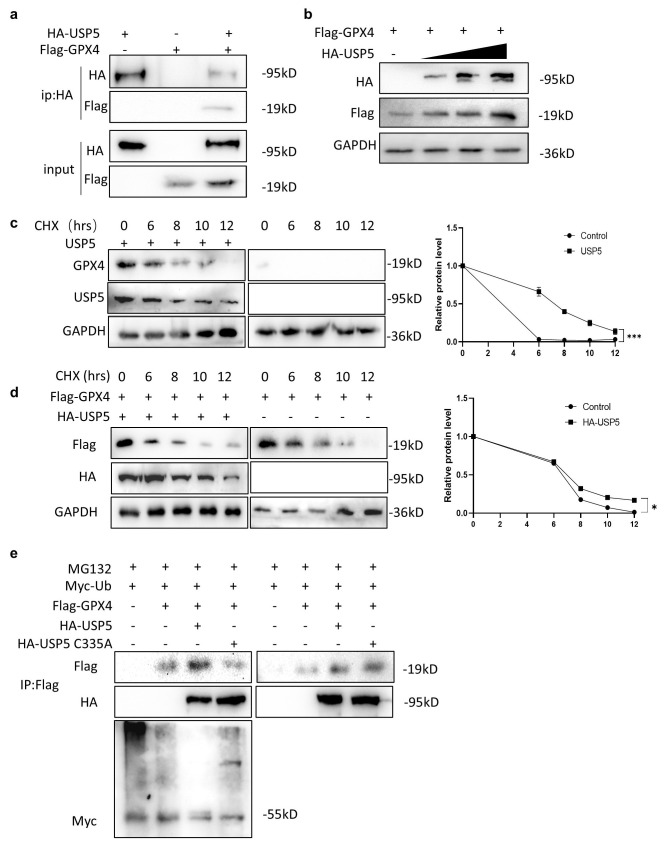
USP5 interacts with and stabilizes GPX4. (**a**) Exogenous interaction between USP5 and GPX4 was confirmed in HEK293T cells through coimmunoprecipitation assays. (**b**) HEK293T cells were transfected with FLAG-GPX4 and increasing doses of HA-USP5 (0, 100, 200, or 500 ng), followed by immunoblot detection of protein expression levels. (**c**) Protein stability was conducted using CHX (50 µg/mL) for the indicated times, with GPX4 and USP5 degradation profiles monitored by Western blot using GAPDH as internal control. (**d**) CHX-based (50 µg/mL) protein stability experiments were performed in HEK293T cells expressing FLAG-GPX4 with or without HA-USP5, with GAPDH serving as normalization control. (**e**) Ubiquitination assays were carried out in HEK293T cells transfected with FLAG-GPX4 and Myc-Ub, in combination with either wild-type HA-USP5 or its catalytically impaired C335A mutant, following 6-hour proteasome inhibition with MG132 (10 µM). The data are representative of 3 independent experiments. Statistical significance was analyzed by ANOVA or Student’s *t* test. * *p* < 0.5, *** *p* < 0.001.

Through the above results, we next investigated the relationship between USP5 and GPX4. Coimmunoprecipitation confirmed the interaction between USP5 and GPX4 (Figure 4a). Considering that USP5 is a deubiquitinating enzyme, we investigated whether USP5 affects the stability of GPX4. The result shows that GPX4 protein levels increased when USP5 was overexpressed. What is more interesting is that this increase occurred in a dose-dependent manner (Figure 4b). To establish the effect of USP5 on GPX4 stability, cycloheximide (CHX) chase experiments were conducted to verify the time course of GPX4 degradation. Genetic ablation of USP5 markedly accelerated GPX4 degradation, as evidenced by its shortened half-life (Figure 4c). Complementation studies demonstrated that USP5 transfection into cells effectively stabilized GPX4 (Figure 4d). To elucidate the underlying mechanism. We next investigated whether USP5 could reduce GPX4 ubiquitination. The results revealed that USP5 overexpression significantly decreased polyubiquitinated GPX4 levels. However, overexpression of USP5 C335A (catalytic-inactive mutant) abolished this reduction (Figure 4e).

These experimental data collectively demonstrate that USP5 physically interacts with GPX4 and post-translationally regulates its stability through deubiquitination.

## 4. Discussion

Bladder cancer is a common malignant tumor with high morbidity and mortality. Current treatment methods have limitations, and there is an urgent need to explore new therapeutic targets. Our study focused on the role of USP5 in bladder cancer and its mechanism, aiming to provide new ideas and targets for the treatment of bladder cancer.

Our results demonstrated that USP5 deficiency significantly inhibits the proliferation and colony formation of T24 bladder cancer cells. These findings are consistent with previous studies showing that USP5 is overexpressed in various cancers, including bladder cancer, and promotes tumor growth and progression. The ability of USP5 to increase cell proliferation may be attributed to its role in stabilizing key proteins involved in cell cycle regulation and apoptosis. For example, USP5 has been shown to interact with and stabilize proteins such as p53, FoxM1, and β-catenin, which are critical for cancer cell survival and proliferation. Our findings further support the notion that USP5 plays a crucial role in bladder cancer progression by promoting cell proliferation and tumor formation. Ferroptosis, a new type of cell death discovered in 2012, is different from apoptosis and necrosis. Ferroptosis has a unique role in anticancer therapeutic strategies. Many studies have shown that ferroptosis is associated with the metastasis, treatment, and prognosis of bladder cancer [25]. Therefore, accurate identification of ferroptosis biomarkers in patients with bladder cancer is expected to lead to subsequent molecular-targeted precision therapy [26]. MDA is one of the main products of membrane lipid peroxidation, and its content is usually used as an index of lipid peroxidation to reflect the degree of cell membrane lipid peroxidation. Mitochondrial ROS are important not only for the induction of apoptosis but also for the induction of ferroptosis. Therefore, the ferroptosis process is often accompanied by increases in the MDA and ROS contents [27]. We found that the Fe^2+^, MDA, and ROS contents were increased in bladder cancer cells when USP5 was depleted. These results suggest that the loss of USP5 can promote ferroptosis in bladder cancer cells. To verify this correlation, we added the ferroptosis inducer erastin to bladder cancer cells for further experiments. Erastin, a ferroptosis inducer first discovered in 2003, was found to exhibit considerable lethality in human tumour cells harbouring mutations in the HRAS, KRA, and BRAF oncogenes [28]. Our experimental results revealed that USP5 deletion caused a more significant increase in the content of the ferroptosis markers Fe^2+^, MDA and ROS in bladder cancer cells after the addition of erastin. Thus, USP5 deficiency promotes ferroptosis in bladder cancer cells.

The Nrf2-HO-1-GPX4 axis is recognized to play an important role in cellular defense mechanisms because of its critical role in resistance to oxidative stress. Inhibition of antioxidant pathways may lead to increased oxidative stress, increased intracellular iron accumulation, and potential induction of ferroptosis [29]. GPX4 is a central regulator of ferroptosis as well as erastin-induced ferroptosis [30]. The pharmacological induction of ferroptosis mainly involves the inhibition of the SLC7A11-GPX4 pathway [31]. The extrinsic ferroptotic pathway involves the use of erastin, sulfasalazine, or sorafenib to inhibit SLC7A11-mediated cysteine uptake in the cell membrane and subsequent GSH synthesis in the cytoplasm [32]. First, we analyzed the correlation between USP5 and GPX4 in bladder cancer. The results revealed a positive association between USP5 and GPX4 RNA levels in bladder cancer tissue. We further verified that USP5 deficiency led to a significant reduction in GPX4 protein levels and that the overexpression of USP5 stabilized the GPX4 protein. Studies have shown that the knockdown or pharmacological inhibition of USP8 increases the sensitivity of colorectal cancer cells to ferroptosis and that USP8 interacts with GPX4 and prevents GPX4 protein degradation by affecting GPX4 deubiquitination [18]. Therefore, we performed a coimmunoprecipitation assay to verify the physical association between USP5 and GPX4. To determine whether USP5 stabilizes the GPX4 protein by deubiquitination, CHX tracking and deubiquitination assays were performed, and the results suggested that USP5 stabilized the GPX4 protein by inhibiting its ubiquitination. These findings suggest that USP5 plays a protective role in bladder cancer cells by stabilizing GPX4 and preventing ferroptosis.

Ferroptosis is a promising target for cancer treatment. GPX4 overexpression serves as a biomarker for aggressive tumor behavior, while its inactivation-driven ferroptosis offers a mechanism-guided avenue for anticancer interventions. Recent studies have demonstrated that the use of GPX4 inhibitors such as RSL3, ML210, ML162, and FIN56 can effectively induce ferroptosis in cancer cells [33,34,35,36]. However, the translational potential of GPX4 inhibitors in vivo is significantly hindered by non-specific target engagement, which may lead to dose-limiting toxicities and compromise therapeutic safety [37]. To date, clinical deployment of these agents remains unreported, with most investigations confined to preclinical studies. In contrast to the extensive exploration of GPX4 inhibitors, a number of studies have explored the factors influencing GPX4 protein stability and its dynamic homeostasis. USP8 stabilizes GPX4 by suppressing its ubiquitination-dependent degradation, thereby attenuating ferroptosis sensitivity in tumor cells, driving tumorigenesis, and inducing resistance to PD-1/PD-L1 blockade [24]. CST1 inhibits ferroptosis and promotes gastric cancer metastasis by regulating GPX4 protein stability [38]. Our findings suggest that targeting USP5 could increase ferroptosis and suppress tumor growth in bladder cancer. As a cysteine protease, USP5 contains a catalytic cysteine box with a nucleophilic thiol group. This chemically reactive site renders USP5 more feasible for pharmacological modulation compared to E3 ligases, which exhibit structural complexity limiting drug development. Given USP5’s central role in maintaining cellular homeostasis and its dysregulation across oncological, neurological, and systemic diseases, pharmacological targeting of USP5 has rapidly evolved into a strategic focus for mechanism-guided drug discovery [37]. This is particularly relevant given the limited therapeutic options currently available for advanced bladder cancer. Inhibitors of USP5 or its downstream targets, such as GPX4, could be developed as novel therapeutic agents for bladder cancer. Moreover, our study highlights the importance of the ubiquitin–proteasome system in regulating ferroptosis. The deubiquitinating activity of USP5 appears to be critical for maintaining GPX4 stability, as evidenced by the fact that the catalytically inactive mutant USP5 C335A failed to reduce GPX4 ubiquitination. These findings suggest that the enzymatic activity of USP5 is essential for its role in regulating ferroptosis, further supporting the potential of USP5 as a therapeutic target.

**Figure 5:**
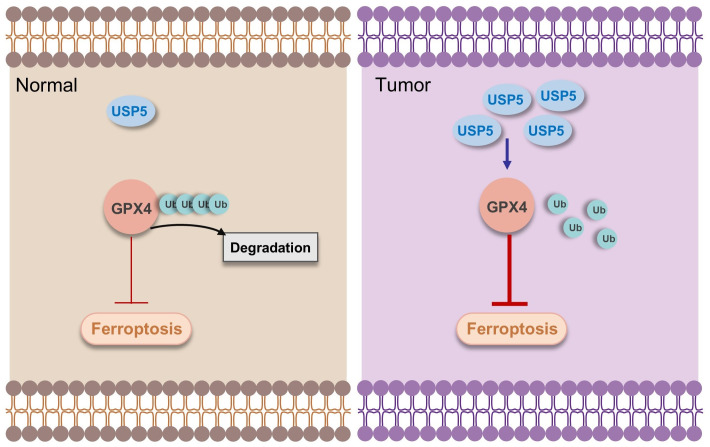
Mechanistic diagram. In normal cells, USP5 is expressed at low basal levels, and GPX4 is subjected to ubiquitin–proteasome-mediated degradation, thereby maintaining a relatively low capacity for ferroptosis suppression. In contrast, in tumor cells, elevated USP5 expression attenuates GPX4 ubiquitination, resulting in enhanced stabilization of GPX4 and consequently increased resistance to ferroptosis.

Based on our experimental evidence, we propose a mechanistic framework wherein USP5 enhances GPX4 stability by suppressing its ubiquitination-mediated degradation, ultimately diminishing tumor cell susceptibility to ferroptosis and facilitating oncogenic progression (Figure 5). While our study provides valuable insights into the role of USP5 in bladder cancer and ferroptosis, several limitations should be addressed in future research. First, our findings are based primarily on in vitro experiments using the T24 bladder cancer cell line. Future studies should validate these results in other bladder cancer cell lines and in vivo models to confirm the generalizability of our findings. Second, the precise molecular mechanisms by which USP5 stabilizes GPX4 and regulates ferroptosis remain to be fully elucidated. Further investigations into the specific ubiquitination sites on GPX4 and the role of other deubiquitinating enzymes in this process could provide additional insights. Third, the universality of the stability of USP5 to GPX4 in other bladder cancer subtypes still needs further validation, and future studies could explore biomarkers for combined USP5 and GPX4 therapy. In conclusion, our study demonstrated that USP5 plays a critical role in bladder cancer progression by promoting cell proliferation and inhibiting ferroptosis through the stabilization of GPX4. These findings highlight the potential of USP5 as a therapeutic target for bladder cancer and provide new insights into the molecular mechanisms underlying ferroptosis regulation. Future research should focus on developing USP5 inhibitors and exploring their efficacy in preclinical and clinical settings, with the ultimate goal of improving outcomes for patients with bladder cancer.

## Figures and Tables

**Table 1:** Sequences of the primers used for RT‒qPCR.

Name	Sequence
GAPDH	Forward GACAAGCTTCCCGTTCTCAGReverse GAGTCAACGGATTTGGTCGT
GPX4	Forward GAGGCAAGACCGAAGTAAACTACReverse CCGAACTGGTTACACGGGAA
NRF2	Forward CTTTTGGCGCAGACATTCCCReverse GACTGGGCTCTCGATGTGAC
HO-1	Forward CAGTGCCACCAAGTTCAAGCReverse GTTGAGCAGGAACGCAGTCTT

## Data Availability

Data is contained within the article. The data presented in this study are available on request from the corresponding author.
